# Maintaining Health in Later Life: Perspectives of Older Indian Migrants in Melbourne, Australia

**DOI:** 10.1002/hpja.70209

**Published:** 2026-06-23

**Authors:** Simran Sandhu, Sabrina Gupta, Raelene Wilding

**Affiliations:** ^1^ La Trobe University Melbourne Australia

**Keywords:** cultural practices, emigrants and immigrants, health behaviour, health practices, healthy ageing, preventive health services, social determinants of health

## Abstract

**Issues Addressed:**

Little is known about how older migrants maintain their health, despite health promotion's emphasis on strengthening everyday capacities for well‐being. Older Indian migrants are one of Australia's fastest‐growing ageing populations, yet existing research has focused predominantly on barriers, disease management and service access. As a result, the health‐promoting strategies older migrants already use remain largely invisible in policy and practice. This study addresses this gap by exploring the practices that older Indian migrants in Melbourne employ to maintain their health.

**Methods:**

A qualitative study was conducted using semi‐structured individual and focus group interviews with 55 India‐born adults aged 50+ (32 temporary and 23 permanent migrants) living in Melbourne, Australia. Interviews were undertaken in English, Hindi and Punjabi.

**Results:**

Participants identified interconnected practices underpinning everyday health maintenance, including reliance on home remedies; culturally familiar yet adaptive dietary and physical activity routines, including walking; domestic labour and group activities. Community and family networks facilitated motivation and support, while gendered household roles constrained women's capacity for rest and engagement in structured physical activity.

**Conclusion:**

This study found that older Indian migrants sustain health through home remedies, culturally embedded dietary routines, physical activity and a strong social network. These practices reflect a strengths‐based orientation to well‐being, drawing on intergenerational knowledge and community support to maintain health in later life.

**So What:**

Health‐promotion strategies should work with, rather than replace, the culturally grounded practices older Indian migrants already use. Culturally considerate, family‐centred health advice; intergenerational activities and digital media partnerships may enhance healthy ageing.

## 1 Introduction

It is now widely recognised that health promotion efforts must reflect the needs and expectations of older adults from multicultural and multilingual backgrounds [1, 2, 3, 4, 5, 6]. Australia's population is ageing [7], and older adults from South Asian backgrounds, particularly those born in India, are among the fastest‐growing ageing communities [8]. The India‐born population now constitutes Australia's second‐largest overseas‐born group (845 800) [9]. By 2056, the number of older Australians born in Asia is projected to be five times higher than in 2021, making up almost one‐fifth of the national older population [8]. While research has demonstrated that migrant populations face more barriers to healthcare access than their Australian‐born counterparts [1, 6, 10, 11] and engage with health services through culturally informed practices [3, 10, 11, 12], little is known about the health practices of older Indian migrants and how they stay healthy. This gap is particularly relevant to health promotion, which emphasises understanding and supporting the strengths and everyday capacities that individuals and communities draw upon [1, 4, 13], consistent with salutogenic perspectives in health promotion that foreground the resources and capacities enabling people to maintain health rather than focusing solely on risk and disease [14]. This further aligns with the view of health as ‘a resource for living that permits people to lead a meaningful life’ [15].

A small body of research has begun to consider broader perceptions of health among South Asian communities. Subedi et al. [16], for example, found that South Asian migrants in Melbourne, Australia, emphasised holistic understandings of health, expressing perspectives like ‘healthy body, healthy mind’, ‘health is wealth’ and ‘health means everything’. These findings highlight the cultural salience of health and well‐being within South Asian contexts. However, much of this knowledge has considered the South Asian population as a homogenous group, concealing the heterogeneity of multiple languages, cultural and religious norms and health belief systems within it. The present study sought to address this by focusing specifically on older Indian migrants as a distinct subgroup while attending to diversity within this group across migration status, gender, age, living arrangements and language, recognising that these dimensions shape how health practices are understood and enacted in everyday life.

The studies conducted on Indian communities in Australia have additionally examined a range of health experiences, such as how this population manages chronic illness [10, 17], sociocultural influences of physical activity [10, 18] and how pain is understood and managed [19]. However, these studies have largely focused on younger or mid‐life cohorts and have emphasised barriers, illness experiences and structural challenges rather than the everyday health practices of older Indian migrants.

Critiques of the Australian healthcare system further underscore the relevance of examining these gaps. Scholars argue that the selective nature of the health system places limited emphasis on culturally respectful care and fails to address structural inequalities that influence health outcomes [20]. These concerns are particularly pertinent for the older Indian population in Australia, which reflects diverse migration trajectories. This group includes permanent migrants who may have arrived through skilled pathways and are ageing in place [21], as well as late‐life migrants who arrive on permanent or temporary visas for family reunification or to provide or receive care [22]. Length of residence is recognised as an important contextual factor in migrant health, with longer duration of stay generally associated with greater health system familiarity, health literacy and service utilisation [10, 16]. Yet this relationship is not linear; older migrants who arrive later in life may bring decades of established health practices that persist alongside, rather than being displaced by, adaptation to the host country context. Projections indicate that the permanent India‐born population will continue to age, with the median age rising from 35.7 to 41 years in the coming decade [23], and temporary migration among older adults is also likely to increase as families navigate transnational responsibilities and visa constraints [21].

Indian migrants are additionally known to face elevated risks of chronic diseases [12], and these risks are amplified in later life by age‐related conditions such as functional decline and increasing dependency [7, 24]. For both temporary and permanent older migrants, these vulnerabilities intersect with unfamiliar healthcare systems, differing expectations of care and culturally embedded understandings of health and well‐being [1, 16, 19, 20].

In this context, adopting a strength‐based perspective becomes essential. Accordingly, this study is theoretically grounded in Antonovsky's salutogenic framework [14], which shifts attention from the causes of disease to the resources and capacities that enable people to maintain health and well‐being. Central to this framework are three components: comprehensibility (the ability to make sense of one's circumstances), manageability (the perceived availability of resources to cope) and meaningfulness (the sense that life has purpose and that challenges are worth engaging with). Taken together, these constitute a sense of coherence, which Antonovsky proposed as a key determinant of health across the life course [14, 25, 26]. This framework oriented the study towards identifying the everyday practices, resources and routines that older Indian migrants draw upon to maintain their health, which can provide critical insights for developing preventive and culturally responsive health‐promotion strategies. Rather than focusing solely on barriers or service gaps, such an approach foregrounds the capabilities and forms of knowledge that support well‐being in later life. Accordingly, this study turns to a foundational question: What do older Indian migrants living in Melbourne, Victoria, do to stay healthy?

## 2 Methods

### 2.1 Study Design

This study used a qualitative design to explore the everyday health‐maintaining practices of older Indian migrants living in Victoria, Australia. A qualitative approach was well suited to this inquiry because it enabled participants to describe, in detail, the routines, strategies and resources they draw upon to stay healthy [27]. Recognising that older migrants may feel more comfortable speaking about health within familiar social configurations, the study offered three formats for participation: individual interviews, focus group discussions and family group interviews. Providing this choice supported culturally appropriate engagement by accommodating preferences regarding privacy, gender norms, collective decision‐making and the presence of family members.

The design prioritised flexibility, relational rapport and cultural sensitivity [28] considerations, which are particularly important for an older cohort whose health practices may be embedded within long‐standing habits, transnational caregiving roles and community networks. This approach aligned with calls within migrant health research to engage methods that capture diversity of experience and acknowledge the relational and contextual nature of health practices among ageing migrant communities [28].

### 2.2 Study Participants and Recruitment

The study was conducted between August 2023 and August 2024 in Victoria, Australia, where approximately 371 901 people identify as Indian [9]. Eligibility criteria included self‐identifying as Indian, being aged 50 years or older [10], residing in Victoria on a permanent or temporary visa, and being able to participate in English and/or Hindi. Recruitment occurred through Indian community organisations, seniors' associations, local councils, religious institutions and informal networks [29]. Flyers, bilingual information sheets and snowball sampling were used to engage participants with varying levels of connection to formal community groups.

Relational trust was prioritised in recruitment. The primary author's existing community relationships, combined with informal endorsements from respected community members, supported culturally appropriate engagement [30]. All recruitment materials emphasised confidentiality, voluntary participation and the option to choose the preferred interview mode.

A total of 55 participants took part in the study, including 32 temporary migrants and 23 permanent migrants (including Australian citizens). Table 1 presents the demographic characteristics of participants. The majority were aged between 61 and 70 years (*n* = 33), with the largest group falling in the 66–70 age range (*n* = 18). There were slightly more male (*n* = 31) than female (*n* = 24) participants. In terms of migration status, the majority were residing in Australia on a visitor or temporary visa (*n* = 32), with the remaining participants holding Australian citizenship (*n* = 15) or permanent residency (*n* = 8). Almost all participants lived with family members (*n* = 52), reflecting the predominantly family‐based migration trajectories of this cohort.

**TABLE 1:** Demographic characteristics of participants.

Characteristics	Category	*n*
Age group (years)	18–49	1
	50–55	2
	56–60	4
	61–65	15
	66–70	18
	71–75	12
	76–80	3
Gender	Male	31
	Female	24
Migration status in Australia	Visitor/temporary visa	32
	Australian citizen	15
	Australian permanent resident	8
Living with family members	Yes	52
	No	3

For the purposes of this study, temporary migrants refer to those residing in Australia on time‐limited visas, such as parent and visitor visas. Visa status was treated as an analytic category, enabling systematic comparison across groups. This distinction matters in the Australian context, where visa status determines access to Medicare (Australia's universal public health insurance scheme), the Pharmaceutical Benefits Scheme (PBS, which subsidises the cost of prescription medicines) and publicly funded aged‐care services, entitlements generally unavailable to temporary visa holders. Although many older Indian migrants arrive on temporary visas to join adult children, several remain for extended periods through renewals or transitions to permanent residency, with access increasingly shaped by caps, processing times and financial thresholds [21, 22, 24]. Where relevant, these structural conditions are noted in the interpretation of findings. Sample size was guided by theoretical saturation, defined as the point at which no new information or practices emerged after repeated interviews [31].

### 2.3 Data Collection

Participants chose between individual interviews, focus groups or family group interviews. The primary author, who identifies as Indian, conducted all interviews and adopted a relational approach to establish rapport and facilitate open conversation [28]. Participants were encouraged to speak in English, Hindi or Punjabi, according to their preference.

Data collection comprised two family group interviews, five individual interviews and nine focus group discussions. Sessions ranged from 30 to 90 min. All interviews were audio‐recorded with consent. Interviews conducted in Hindi were translated into English by the primary author and reviewed by Author 2 to ensure accuracy and preserve cultural nuance [32].

Ethics approval was obtained from the La Trobe Human Research Ethics Committee. Written informed consent was obtained from all participants, who were reminded of their right to withdraw up until 4 weeks following data collection. All data were anonymised and stored securely in accordance with COREQ guidelines [33].

### 2.4 Data Analysis

Data were analysed thematically using an inductive approach supported by NVivo 1.7.2 [34]. The analytic process was informed by Antonovsky's salutogenic framework [14], which oriented coding and thematic development towards the everyday resources, capacities and practices through which participants maintained health, specifically attending to how participants made sense of their circumstances (comprehensibility), mobilised available resources (manageability) and drew meaning from their roles and relationships (meaningfulness). Open coding identified descriptions of health routines, home practices, dietary behaviours, physical activity, use of traditional or complementary practices and the role of family, friends and community. Codes were then organised into broader themes through iterative team discussions, memo writing and repeated engagement with the data [28].

Multilingual analysis was essential. Frequent switching between Hindi, English and Punjabi required careful attention to cultural nuance, tone, idioms and emotion during transcription and translation [34]. Translation was treated as an interpretive process central to meaning‐making and representation, consistent with qualitative research standards [32]. Collaborative review by bilingual team members enhanced accuracy and rigour [32].

To strengthen credibility, preliminary themes were returned to a small number of original study participants, including a community leader who had taken part in the research, for feedback and validation (member checking). Themes were shared in de‐identified, aggregated form to protect the confidentiality of all participants. Reflexivity was embedded throughout the study. The researcher's shared cultural background supported rapport but required continual attention to avoid assumptions [32]. The analysis team included members with Indian and non‐Indian backgrounds, allowing for diverse perspectives and reducing potential bias.

## 3 Results

The thematic analysis generated four interrelated areas that characterise how older Indian migrants maintain their health in everyday life. Participants described drawing on a range of traditional and complementary approaches to support well‐being (Section 3.1), engaging in food‐based routines and culturally grounded dietary practices (Section 3.2), incorporating physical activity into daily life (Section 3.3) and relying on community, family and social networks to sustain motivation for engaging in lifestyle‐related behaviours and connectedness (Section 3.4).

### 3.1 Traditional and Complementary Approaches to Health

Traditional and complementary approaches were central to how participants maintained their health in everyday life. Across interviews, participants described relying on familiar home‐based remedies as their first response to minor symptoms. These practices were part of daily routines and were viewed as effective, accessible and culturally embedded. As one female participant explained,

Yes, we do things like that in our routine. For example, if someone has a cold or cough, we make बेसन का शीरा (a hot syrup made from gram flour, ghee, milk and spice) … and give it to them hot at night‐ it helps a lot.

Participants commonly described a staged approach in which they first tried home remedies before considering formal healthcare. One female participant noted,

पहले तो मैं घर के अल्ले‐छल्ले करूंगी (first I will do home remedies), दादी मां के नुस्खे (grandmother's techniques)… I will do first. If it doesn't resolve after that I will then go to my GP… You have to give it one or two days for anything to resolve.

For many, this sequence was routine and shaped the way they responded to everyday illness.

Accounts emphasised the familiarity and trust associated with these remedies, which were often learned from elders and used throughout life. One male participant reflected,

Yes, especially among us, we do that more. We try what our elders have told us; we use our own knowledge. Headache? Do this. Stomach‐ache? Eat that… We grew up in an environment like that in India, after all. And we trust that.

Such practices were described as part of how participants had always looked after their health, including after migration.

Several participants also incorporated Ayurvedic medicines into their regular health routines. These were sourced from India, either during travel or sent through family members. One female participant stated,

I just started Ayurvedic medicine, and I'm taking that [for knee pain]. It has given me a lot of relief… I started taking Ayurvedic medicine from India, which has been beneficial. Yes, I get them (from India) when I go there. I also get them couriered [to Australia].

Ayurveda was described as helpful, particularly when biomedical treatments were perceived as less effective.

### 3.2 Adapting Dietary and Medicine‐Taking Practices

Participants described food and dietary practices as central to managing their health in Australia. Several reflected on how the visibility of Indian ingredients in the wider Australian food landscape had shifted over time, noting that this allowed them to openly use ingredients they valued for their well‐being, without fear of judgement. As one female participant expressed,

I used to be so embarrassed when frying garlic and ginger, and now they have turmeric lattes and ghee in cafes. It feels like what we used to hide is now in fashion – and people are finally understanding that this stuff works.

Some, on the other hand, discussed adapting their diets as part of ageing and settling into new routines after migration. Many described reducing certain foods, moderating portion sizes and incorporating healthier habits into their everyday life. One male participant explained,

I avoid fatty food and all of that… sometimes I'm naughty. I'll have a whole piece of cake… but overall healthy eating. It's a routine now. It's like brushing your teeth in the morning? Yeah, you have to do it.

The process of dietary adaptation was described as gradual, particularly among temporary migrants who had settled in Australia later in life and were adjusting long‐standing routines to a new environment. This was illustrated in a focus group exchange:

Speaker 1 (female) It takes time to adapt.

Speaker 6 (male) Yes, it does.

Speaker 5 (male) And we've cut back on the medicines and food habits that we had in India.

Speaker 6 (female) ‘Yes, we used to take a lot more medicine in India.’

Participants explained that the kinds of routine over‐the‐counter medicines and habitual food practices they commonly relied on in India were used far less in Australia. For many, this reflected a shift in what felt suitable or necessary for maintaining health in their current circumstances.

Participants who regularly moved between India and Australia described a need to adjust their diet across contexts. One female temporary migrant explained,

So the diet that I am doing in India… here like even talking about the taste of things… that even if we want to do a diet here, I don't understand anything [ingredients, groceries etc.] but it is okay, I can manage that, there are things available here as well, it is more about accepting it.

For these participants, maintaining health required modifying long‐standing dietary habits in ways that fit their circumstances in Australia, including negotiating differences in taste, routine and the availability of familiar foods.

Older adults who migrated later in life also described making more substantial changes to their dietary identity as part of adapting to the Australian context. One female participant shared:

For survival, you have to adapt. Earlier I was completely vegetarian, but here I eat everything. Before, I didn't eat on Tuesdays, not even onions, and I used to wear yellow clothes that day. Now I don't do that… So you have to adapt.

The Tuesday reference reflects a Hindu practice involving weekly food‐based and colour‐based observances [35]. Participants noted that maintaining such routines felt difficult within Australian lifestyles, work patterns and household arrangements, making adaptation a practical part of staying healthy in later life.

### 3.3 Physical Activity and Exercise

Participants described movement and daily physical activity as an important part of staying healthy in Australia. Many emphasised the need to remain active and socially engaged, even when unwell. One female participant explained, ‘No matter how sick we are, we don't stop going out. We go to the club, meet others and keep moving around’. Another male participant observed, ‘I've realised the more you move the more you stay happy here [in Australia]’. Movement was therefore described as closely connected to emotional well‐being, motivation and maintaining a sense of balance in daily life.

Participants engaged in a range of physical activities, from neighbourhood walks to structured exercise. Some described regular walking routines embedded into their mornings and evenings: ‘We go for a walk in the morning and then again in the evening’.

Others described more structured forms of exercise, including the use of fitness trackers and weekly sports. One male participant noted,

I walk around 10,000 steps every day. I record everything and twice in a week I play badminton with my group around 10–12 people. We are very competitive.

Among temporary migrants, particularly women who arrived later in life, physical activity was often shaped by the demands of household work, caregiving and expectations tied to gendered roles. These participants described wanting to rest or take time for exercise but finding it difficult to do so. One participant reflected, ‘It's just that… it's not that I don't live comfortably; sometimes I feel I should rest, but I don't get the time… I become a bit restless’. Another added, ‘I think maybe I should finish the housework… even though there isn't much left, it still feels like it is there’.

These reflections pointed to how domestic labour functioned as women's primary form of exercise but also restricted their capacity to engage in planned exercise or to take restorative breaks.

### 3.4 Community, Family and Social Networks

Social connections played a central role in how participants maintained their health in later life. For many, particularly temporary migrants who had relocated to Australia in older adulthood, regular involvement in community groups, clubs and friendship networks was described as essential not only for companionship but for staying active, informed and emotionally supported.

Participants frequently emphasised the importance of their local seniors' club for accessing reliable health information and navigating the Australian health system. One male temporary migrant shared, ‘We receive all our health information from the club. Even the COVID vaccines, we got them through here’. Another male participant described the structured health sessions facilitated through the club,

Since we've formed this club, we've gotten a lot of help. X Health organises sessions every three to four months. They even bring interpreters so people can fully understand the information in Hindi.

These sessions addressed seasonal illnesses, preventive care and health risks relevant to older adults, positioning the club as both a social hub and a trusted health‐promotion setting.

Peer support within these networks was also emphasised. Participants described relying on friends for practical and emotional assistance, particularly when adult children were unavailable or lived elsewhere:

‘If the kids aren't here, we can go [to the doctor] with friends. Friends are always around to help out’. This support was often described as reciprocal as one participant shared, ‘Supporting each other is really good here [in the club]’. (Male)

The sense of belonging produced through these networks was framed as integral to feeling well. One male participant remarked humorously on the vitality that club participation provided: ‘He's young because he joined Club X. Otherwise, he'd be walking around with a cane by now’.

Informal social routines such as walks in the park, attending community events or meeting friends for tea or coffee were similarly woven into participants' everyday lives, particularly among permanent migrants who described more settled and predictable daily rhythms. As one female participant shared: ‘I do more cooking, gardening, meet with friends and have coffee meetings… Now I do, because I'm used to it here [ Australian lifestyle]’.

Family relationships also shaped well‐being, particularly for those living in multigenerational households. One female participant described the comfort and rhythm of shared daily life:

I spend the whole day with the joint family, so my grandchildren are there, and spending time with them keeps me occupied. I also do household chores, take care of the kitchen, watch Indian TV, use my phone, and sometimes I pray. Time passes by. I feel good about it. I'm not very worried about such things that I am unable to spend my time. I'm happy that my time is passing well. I don't even realize how time flies.

## 4 Discussion

The practices described in this study broaden prevailing understandings of migrant health in Australia, showing that older Indian migrants' everyday efforts to maintain well‐being extend well beyond what is commonly captured in illness‐focused or barrier‐focused accounts. Much of the existing research on Indian migrants has centred on addressing health access challenges [36, 37], chronic disease management [10, 19], physical activity constraints [10] or health beliefs [38]. While this scholarship has been essential in identifying inequities, it leaves under‐explored the everyday forms of knowledge, care and adaptation through which older migrants actively sustain health outside the clinic. The accounts presented here demonstrate that older Indian migrants do not approach health as passive recipients within a new system but as individuals drawing on long‐standing explanatory models, intergenerational skills and culturally grounded practices that continue to guide self‐care in later life.

Across the interviews, home remedies and Ayurvedic principles featured as the most trusted first responses to discomfort or minor illness. Participants' use of home remedies before consulting a GP echoed Ahmad et al.'s [18] findings among Indian migrants who trialled home remedies or Ayurvedic treatments before initiating diabetes medication. Rather than signalling avoidance of biomedicine, these actions align with Rao's [39] ‘hierarchy of resort’, wherein the first therapeutic step reflects familiarity, safety and collective wisdom. Ayurvedic remedies were also commonly drawn upon, grounded in the belief that they were natural and therefore unlikely to cause side effects, a perception that has been documented in previous research on chronic illness management in India [12]. For many older migrants, especially temporary visa holders excluded from Medicare, Australia's universal health insurance scheme, these practices also provided a financially and emotionally manageable way to observe symptoms and decide whether escalation was necessary. Permanent migrants, by contrast, integrated these routines with transnational health strategies, including replenishing Ayurvedic supplies during visits to India or consulting long‐known clinicians in India for reassurance. These differences show that home‐based care is shaped as much by structural conditions as by cultural preference.

Food practices offered another insight into how older migrants maintained their health in Australia. Participants described using familiar ingredients and routines to support well‐being, noting that the growing visibility and availability of Indian foods in the Australian food environment made it easier to continue practices they valued. This accessibility reflected broader processes of bi‐directional acculturation, whereby migrant food practices have become increasingly recognised and incorporated within mainstream food systems, rather than remaining confined to ethnic enclaves. Like Porqueddu's [40] observation that migrant food routines are sustained through everyday domestic practice, participants' accounts showed that these habits were maintained not for symbolic reasons but because they were experienced as effective and familiar. At the same time, many described gradual dietary changes as part of ageing and settling into new routines, such as reducing fatty foods or moderating portion sizes, echoing findings from Gupta et al. [41] that migration prompts shifts in eating patterns among Indian migrants.

A gendered dimension also emerged in these accounts. Women were more likely to articulate detailed knowledge of traditional food‐based remedies and everyday health practices, often positioning themselves as the primary custodians of this knowledge within the household. Their narratives reflected long‐standing caregiving roles, in which responsibility for preparing foods and determining appropriate remedies formed part of their role. Through these practices, women played a key role in transmitting health‐related knowledge across generations, sustaining everyday modes of care that were embedded in family life rather than formal health systems.

Temporary migrants, particularly those who arrived later in life, spoke of adapting long‐standing habits as they negotiated new tastes, ingredients and household rhythms. Some also noted reducing medicines and food‐based practices they relied on in India, reflecting shifts in what felt suitable or necessary in the Australian context—an adaptation process consistent with findings that dietary behaviours among South Asian migrants are shaped by changing environments and practical constraints [16]. Permanent migrants described more settled dietary routines, reflecting greater acculturation to the Australian food environment, consistent with research showing dietary adaptation among South Asian migrants is incremental rather than abrupt [41]. These differences suggest dietary adaptation is shaped not only by cultural preference but also by degree of acculturation and structural conditions associated with visa status.

Movement and physical activity were described not as medical directives but as emotionally grounded practices that kept life meaningful. Statements such as ‘the more you move the more you stay happy here’ show that physical activity was tied to mood, social connection and coping with the demands of migration. This resonates with the finding that movement among Indian migrants is understood holistically rather than purely physiologically and is closely tied to ethnic identity and social interaction [10]. At the same time, the gendered dimension was striking. Temporary migrant women who migrated later in life described domestic labour as both their primary form of movement and a constraint on rest or structured exercise. Their reflections, such as ‘I should rest, but I don't get the time… I become a bit restless’, illustrates how physical activity is entangled with gendered expectations of productivity, caregiving and moral responsibility, alongside feelings of guilt associated with taking time for oneself, as noted in research on South Asian women's engagement with physical activity [10]. For temporary migrants more broadly, physical activity also carried a stronger emotional and preventive dimension, described as a way of maintaining well‐being and staying ahead of health decline in a context where formal healthcare access was limited. For permanent migrants, physical activity was more deeply embedded as an established daily routine, reflecting greater settlement and stability. These accounts show that movement for older migrants cannot be understood without attending to gender, the moral weight of domestic contribution and the structural conditions associated with visa status that shape both the motivation for and accessibility of physical activity.

Community networks emerged as a central resource for staying healthy, particularly among temporary migrants who arrived later in life but also for permanent migrants whose daily rhythms were anchored in stable routines. Participants consistently described their seniors' club not only as a site for receiving reliable information and translated health education but also as a place where they felt energised, motivated and emotionally uplifted. This aligns with World Health Organization (WHO) guidance [42], which recognises migrant community groups as culturally safe environments that support health, well‐being and engagement with care. The emphasis on belonging ‘he's young because he joined the club’ shows how social participation itself was perceived as protective. Karl and Torres [43] similarly note that for ageing migrants, community belonging is central to sustaining well‐being in unfamiliar contexts. In our study, the club's significance was not only about navigating structural gaps such as the Medicare exclusion. It was equally about reducing loneliness, finding purpose, establishing routine and receiving mutual support, especially when family members were busy or geographically distant. The joy, laughter and vitality participants associated with their clubs reflect how social ties operate as a health resource.

Taken together, these practices reflect a coherent pattern that aligns strongly with salutogenic principles. Participants drew on long‐standing explanatory frameworks to interpret symptoms (comprehensibility), used trusted routines such as home remedies, adjusted diets and community groups to maintain control and stability (manageability) and grounded health behaviours in meaningful roles as grandparents, caregivers and active community members (meaningfulness). These culturally embedded practices mirror Antonovsky's [14] notion of generalised resistance resources and align with empirical work showing that a sense of coherence supports well‐being in later life [25, 26]. However, our findings also demonstrated how these resources were not distributed evenly. Visa status shaped access to formal care and transnational support; gender shaped time available for exercise and rest; yet across these differences, participants assembled strategies that sustained coherence and health in their everyday lives.

Although language barriers are often emphasised in research on older migrant groups, this was not explicitly seen as a challenge to staying healthy in our cohort. Participants demonstrated clear awareness of how to stay healthy and actively combined home remedies, dietary adjustments, social routines and biomedical care. Gendered expectations and, for temporary migrants, financial and entitlement constraints shaped how easily these practices could be sustained. Taken together, the findings suggest that the task is not only to address knowledge gaps but also to recognise and build on the existing health practices of older Indian migrants. Designing health promotion that aligns with these culturally grounded routines may support stronger and more sustained engagement in later life.

## 5 Strengths and Limitations

The qualitative design of this study was a key strength, as it enabled detailed accounts of the routines, practices and supports older Indian migrants use to maintain their health. Offering individual, family and group interviews allowed participants to choose a format that felt culturally and personally comfortable, which enhanced the depth and openness of discussions. The inclusion of both permanent and temporary migrants provided a diverse sample with varied migration histories and caregiving roles, strengthening the breadth of perspectives captured. Multilingual data collection across English, Hindi and Punjabi allowed participants to speak in the language that best conveyed their everyday practices and the bilingual review of translations supported accuracy and cultural nuance. Reflexive practice, collaborative coding among team members with Indian and non‐Indian backgrounds and member checking with community representatives further contributed to the rigour of the study.

Several limitations should also be noted. The researcher's insider position, while helpful for rapport‐building, may have contributed to assumptions of shared cultural understanding, potentially limiting deeper probing in some areas. Recruitment through community groups and social networks may have under‐represented individuals who are more socially isolated or disengaged from organised community life. Although multilingual, the study could not capture the full linguistic diversity of the Indian diaspora in Australia and speakers of languages other than English, Hindi and Punjabi may have been excluded. Participation may also have been shaped by gender norms, comfort in group settings and familiarity with qualitative interviews. Additionally, length of stay and period of arrival were not systematically collected as variables, which limits the capacity to examine how duration of residence may have shaped participants' familiarity with the Australian health system or influenced their health practices over time. Future research should consider capturing these variables to enable more nuanced analysis of migration trajectory and its relationship to everyday health practices. These considerations highlight the importance of continued research with older Indian migrants using varied recruitment strategies, multiple languages and a range of methodological approaches to capture the full diversity of experiences.

## 6 Conclusion and Recommendations

This study provides further insight into how older Indian migrants stay healthy, highlighting the ways they actively sustain well‐being through home remedies, food‐based routines, everyday movement and strong community and family connections. These practices reflect a strengths‐based orientation to health consistent with the Ottawa Charter's framing of health as ‘a resource for everyday life’ [44] and align with contemporary health promotion perspectives that emphasise the everyday capacities people mobilise to support well‐being. Rather than depending solely on formal health services, participants drew on intergenerational knowledge, culturally grounded explanatory models and community networks to navigate health in later life.

For health promotion, these findings highlight the importance of working with culturally embedded practices. Netto et al. [45] argue that culturally responsive interventions must begin with the logics through which communities already understand and enact health. Incorporating structured discussions about home remedies, dietary routines and the selective use of Ayurveda into preventive care may support trust, continuity and understanding, thereby strengthening engagement in ways that align with salutogenic principles [14, 25, 26]. The central role of seniors' clubs in participants' accounts further underscores the value of community settings as culturally safe, health‐supportive environments [4, 6, 46]. Strengthening partnerships between health services and seniors' clubs, including the provision of culturally considerate, family‐centred health advice; intergenerational activities and digital media partnerships, may position these community settings as preventive health infrastructure rather than peripheral supports, enabling early, low‐threshold engagement with health information in ways that reflect how older migrants already seek advice and reassurance. However, as noted in the limitations of the study, for those who are socially isolated or disengaged from ethnic networks, stabilising a sense of coherence requires physical and social environments to be deliberately structured to promote salutogenic principles [25]. Therefore, proactive service‐level responses, including outreach through primary care and culturally informed home visiting, may be necessary to ensure their health needs are not rendered invisible within community‐focused health promotion frameworks.

Future research should examine how intersecting factors such as visa status, gendered expectations, socioeconomic position and transnational ties influence the sustainability of these health practices over time. Comparative work with other ageing migrant communities could also reveal whether similar constellations of everyday practices underpin well‐being elsewhere, expanding the evidence base for culturally grounded health promotion. Importantly, in developing such evidence, the heterogeneity inherent within Indian migrant populations must be acknowledged, ensuring that proposed strategies do not inadvertently assume uniform cultural practices or support structures across all groups. Co‐designed studies involving community organisations and health professionals will be essential for developing preventive frameworks that work alongside, rather than attempt to replace, the practices older adults already use to stay well.

## Author Contributions

**Simran Sandhu:** conceptualisation, methodology, investigation (data collection), data curation, formal analysis, writing – original draft, writing – review and editing. **Sabrina Gupta:** conceptualisation, methodology, supervision, writing – review and editing. **Raelene Wilding:** conceptualisation, methodology, supervision, writing – review and editing.

## Funding

This research was supported by a PhD scholarship from La Trobe University.

## Ethics Statement

This study received ethics approval from the La Trobe University Human Research Ethics Committee (Project ID: HEC23285).

## Consent

All participants provided informed consent before participation.

## Conflicts of Interest

The authors declare no conflicts of interest.

## Data Availability

The conditions of human ethics research approval for this project do not permit open sharing of data in order to protect the confidentiality of participants. Any questions about data should be directed to r.wilding@latrobe.edu.au.
